# A Case Series and Review of Hydroxychloroquine Toxicity and Monitoring: How Do We Dose Hydroxychloroquine?

**DOI:** 10.7759/cureus.90681

**Published:** 2025-08-21

**Authors:** Cristine K Arcilla, Myint Thway, Gurjit S Kaeley, Maria Adams

**Affiliations:** 1 Department of Rheumatology, University of Florida College of Medicine – Jacksonville, Jacksonville, USA; 2 Department of Rheumatology, Baptist Medical Center South, Jacksonville, USA; 3 Department of Ophthalmology, University of Florida College of Medicine – Jacksonville, Jacksonville, USA

**Keywords:** cardiotoxic agents, clinical rheumatology, hydroxychloroquine (hcq), hydroxychloroquine level, retinal hydroxychloroquine toxicity, retinal toxicity, rheumatology, therapeutic drug monitoring (tdm)

## Abstract

Hydroxychloroquine (HCQ) is an anti-malarial drug safely used in rheumatologic conditions with its various mechanisms on immunomodulation aside from its impact on cardiovascular health. However, multiple systemic effects, from ocular toxicity to cardiotoxicity, were reviewed and reported within adequate weight-based HCQ doses, challenging the need for alternate HCQ monitoring. We report cases with catastrophic adverse events from chronic HCQ use and supratherapeutic blood HCQ levels while within the recommended weight-based dosing. Cases of supratherapeutic HCQ blood levels have emerged despite adherence to weight-based dosing, indicating the necessity for individualized monitoring. This study aims to highlight the toxicities associated with chronic HCQ use along with the limitations of current weight-based dosing guidelines. This also provides a discussion on the unique pharmacokinetics and pharmacodynamics of HCQ in better understanding proposed HCQ blood level monitoring.

## Introduction

Hydroxychloroquine (HCQ) is one of the most well-tolerated and safest drugs central to many rheumatic and musculoskeletal diseases (RMD). It has been utilized initially as an anti-malarial drug, an analogue of chloroquine, due to its safer profile by acting directly on heme polymerization of the *Plasmodium* parasite [1-3]. It has an organic 4-aminoquinoline compound with an alpha side-chain containing a hydroxyl group [2,3]. This biochemical structure allows for its immunoregulatory and antiproliferative properties. Moreover, these properties are achieved by specifically inhibiting the recognition of nucleic acids by toll-like receptors (TLRs) and preventing major histocompatibility complex (MHC) class II-mediated antigen presentation [2,4]. This pathophysiology is crucial in modulating innate and adaptive immunity utilized in rheumatology. HCQ has been particularly useful since 1894 in cases of cutaneous lupus and was approved for systemic lupus erythematosus (SLE) in the United States beginning in 1955 [1]. Most recent SLE guidelines strongly advocate for HCQ as background therapy and should be optimized unless with adverse effects and contraindications [1]. In addition, HCQ has other mechanisms of action involving lipid and glucose metabolism, cardiovascular protection, hemostasis, photoprotection against harmful ultraviolet radiation, and anti-thrombosis [5-8]. Until recently, anecdotal reports for attenuating viral loads and improving severe respiratory symptoms led to HCQ's utility as a COVID-19 therapy [8-11]. However, this wide coverage of HCQ benefits could also lead to multiple systemic effects, from ocular toxicity to cardiotoxicity, with the crucial need for monitoring. 

After Food and Drug Administration (FDA) approval in 1955, HCQ has been used with dosage recommendations starting from an initial loading dose, followed by a fixed dose of 200-400 mg/day [2]. Over time, dosing was changed to a dose based on the ideal body weight, not exceeding 6.5 mg/kg daily [2,9]. In 2016, the American Academy of Ophthalmology (AAO) recommended using actual body weight with 5 mg/kg or less daily dosing [9,10]. AAO recommended new guidelines as well for HCQ ocular toxicity screening, requiring baseline screening within one year of starting HCQ, followed by annual screening after five years. However, the occurrence of rare but detrimental side effects from chronic HCQ use, including retinopathy and cardiotoxicity, was reported even within adequate actual weight-based dosing [2,9-11]. Major risk factors of retinal toxicity from HCQ observed in studies include use for more than five years' duration (cumulative dose), dose of more than 5 mg/kg of actual body weight, renal disease, and concomitant tamoxifen use [2]. In addition, the screening of corrected QT interval (QTc) prolongation is not employed in the routine monitoring of HCQ cardiotoxicity, particularly for patients with high cardiovascular risk factors. Here, we describe two cases with detrimental adverse events of retinopathy and pro-block and nonischemic cardiomyopathy from chronic HCQ use and an asymptomatic case with supratherapeutic HCQ whole blood level within their recommended actual weight-based dosing.

## Case presentation

Case 1

**Figure 1:**
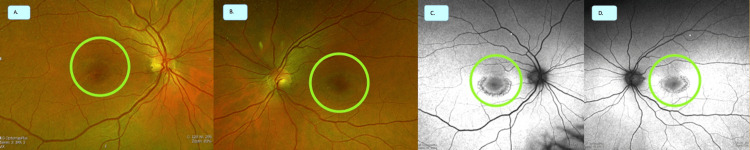
(A and B) Fundoscopy showed right (A) and left (B) classic "bull's eye" maculopathy described as target-like or "bull's eye" pattern due to a well-demarcated concentric zone of atrophy or hypopigmentation surrounding a zone of granular hyperpigmentation of the parafoveal area. (C and D) Fundus autofluorescence showed similar right (C) and left (D) classic "bull's eye" maculopathy with severe central retinal pigment epithelium loss.

**Figure 2:**
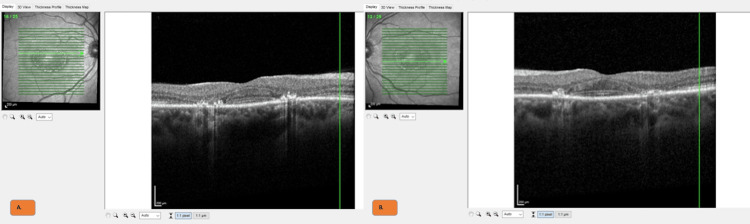
(A and B) Optical coherence tomography revealed the right (A) and left (B) disruption of ellipsoid zone consistent with macular toxicity from long-term hydroxychloroquine use.

A woman in her early 50s with discoid lupus on HCQ for more than 20 years presented for a routine HCQ eye examination. She reported seeing circles with flashing lights inside and complained that her vertical blinds had appeared wavy for the past six months. She was taking HCQ 200 mg twice daily for more than 20 years at a 4.6 mg/kg dose. She was unsure if an optometrist or an ophthalmologist was seeing her for her annual eye examinations. Laboratory findings, as mentioned in Table 1, are notable for a positive antinuclear antibodies (ANA) 1:160 titer with normal complement and creatinine levels. Her visual acuity was correctable to 20/20 oculus uterque (OU), and her color vision testing was 6/8 OU. Her fundus examination showed concentric hypopigmentation around both foveas, consistent with the lesion of classic bull's eye maculopathy (Figure 1). Fundus autofluorescence showed bilateral bull's eye maculopathy as well (Figure 1). Optical coherence tomography (OCT) revealed the classic bilateral disruption of the parafoveal ellipsoid zone (Figure 2). Subsequent Humphrey visual field (HVF) testing revealed bilateral pericentral ring scotomas. Multifocal electroretinography (mfERG) revealed significantly decreased amplitudes and parafoveal macular damage OU, which was also consistent with macular toxicity from long-term HCQ use. HCQ was discontinued immediately. Unfortunately, the patient was lost to ophthalmologic follow-up. 

**Table 1:** Laboratory values of the patient in case 1, notable for a positive ANA 1:160 titer with normal complement and creatinine levels; reference values were included. ANA: antinuclear antibodies; Anti-ds DNA antibody: anti-double-stranded DNA antibody

Laboratory	Value	Interpretation	Reference range
Leukocytes	7.6	Normal	4.5-11×10^3^/cm^3^
Hemoglobin	15.2	Normal	12.0-16.0 g/dl
Platelets	276	Normal	140-440×10³/µL
Blood urea nitrogen	15	Normal	6-22 mg/dl
Creatinine	0.68	Normal	0.51-0.95 mg/dl
Aspartate transaminases	15	Normal	14-33 IU/l
Alanine transaminases	14	Normal	10-42 IU/l
Albumin	4.2	Normal	3.8-4.9 g/dl
Erythrocyte sedimentation rate	14	Normal	≤20 mm/h
C-reactive protein	7.5	Normal	<8.0 mg/l
Urinalysis	Negative	Negative	Negative mg/dl; negative 0-5 HPF
Urine protein/creatinine ratio	77	Normal	24-184 mg/g creat
HIV	Non-reactive	Negative	Negative: non-reactive; positive: reactive
ANA titer and pattern	1:160; nuclear, speckled	Positive	Negative
Anti-ds DNA antibody	1	Negative	Negative ≤4
Complement C3	162	Normal	83-193 mg/dl
Complement C4	41	Normal	15-57 mg/dl

Case 2 

**Figure 3:**
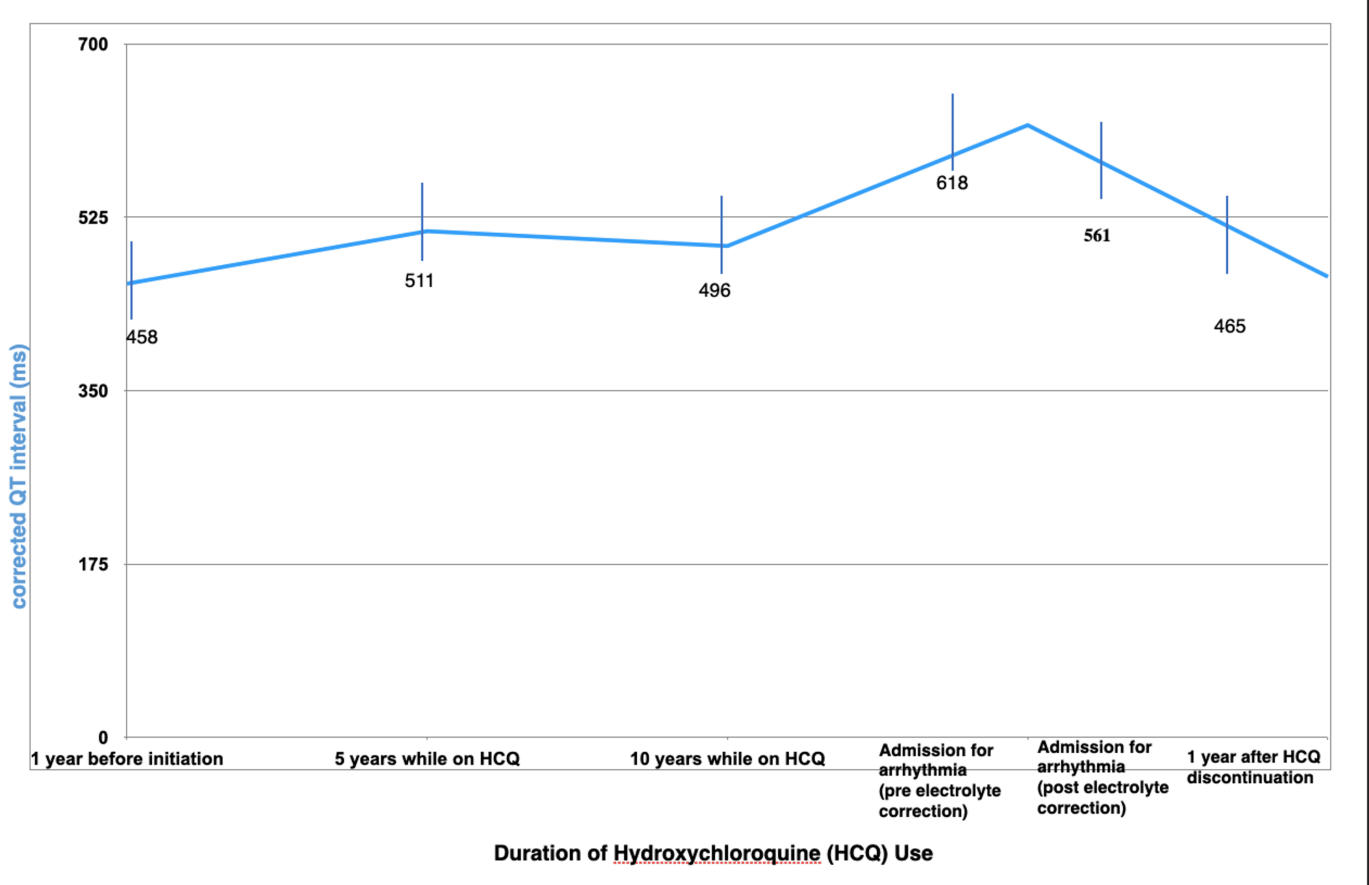
Trend of QTc ranges on electrocardiogram from one year before HCQ initiation to one year after HCQ discontinuation. This reflects normal QTc before HCQ initiation, subsequent QTc prolongation while on HCQ for five and 10 years, respectively, and resolution of QTc prolongation after one year of HCQ discontinuation. Normal QTc ranges are <470 ms for females and <450 ms in males. QTc: corrected QT interval; HCQ: hydroxychloroquine

A woman in her late 40s with seronegative erosive rheumatoid arthritis on HCQ at a 3.6 mg/kg dose consistently for more than 10 years presented for syncope. She was driving home when she experienced lightheadedness, palpitations, and blurry vision before blacking out, causing her to hit her car into a tree. She denies any syncope or seizures in the past and is not on any other QTc-prolonging medications. She has no family history of arrhythmia or sudden death. Examination showed a left periorbital hematoma with no focal neurologic deficits. STAT computed tomography (CT) did not reveal evidence of acute fracture of the head and cervical spine but showed a displaced left naso-orbital ethmoid fracture. CT angiography (CTA) showed no evidence of stenosis. An electrocardiogram (ECG) revealed sinus tachycardia of 119 bpm, without ischemic changes, and a newly prolonged QTc of 618 ms compared to her previous normal baseline ECGs (Figure 3). Laboratory findings, as mentioned in Table 2, are notable for lower potassium and magnesium levels and normal calcium, creatinine, and thyroid-stimulating hormone (TSH). Troponins were normal, while pro-brain natriuretic peptide (pro-BNP) was elevated. Telemetry demonstrated episodes of second-degree atrioventricular block. HCQ was discontinued, and electrolytes were corrected. An electrophysiology study recorded an episode of polymorphic ventricular tachycardia. Echocardiography showed a reduced ejection fraction (EF) of 45-50%. Left cardiac catheterization demonstrated normal coronaries. Cardiac magnetic resonance imaging (MRI) did not show delayed enhancement to suggest an infiltrative disease. Despite correction of electrolytes, QTc prolongation only improved to 561 ms, so a loop recorder was placed before discharge. Serial ECGs were done as outpatient follow-up, showing a normal QTc of 465 ms after a year of HCQ discontinuation, with recovery to normal EF in repeat echocardiography after six months. 

**Table 2:** Laboratory values of the patient in case 2, notable for lower potassium and magnesium levels and elevated pro-BNP; calcium, creatinine, TSH, and troponin levels were normal; reference values were included. TSH: thyroid-stimulating hormone; Pro-BNP: pro-brain natriuretic peptide; CCP: cyclic citrullinated peptide; ANA: antinuclear antibodies; Anti-ds DNA antibody: anti-double-stranded DNA antibody

Laboratory	Value	Interpretation	Reference range
Leukocytes	5.54	Normal	4.5-11×10^3^/cm^3^
Hemoglobin	12.7	Normal	12.0-16.0 g/dl
Platelets	212	Normal	140-440×10³/µL
Potassium	3.1	Low	3.4-4.5 mmol/l
Calcium	9.4	Normal	8.6-10 mg/dl
Magnesium	1.8	Borderline low	1.8-2.6 mg/dl
Blood urea nitrogen	9	Normal	6-22 mg/dl
Creatinine	0.69	Normal	0.51-0.95 mg/dl
Aspartate transaminases	35	Borderline high	14-33 IU/l
Alanine transaminases	32	Normal	10-42 IU/l
TSH	2.3	Normal	0.270-4.200 mIU/l
Troponin	7	Normal	<14 ng/l
Pro-BNP	304	High	0-125 pg/ml
Rheumatoid factor screen	Negative	Negative	Negative
CCP IgG antibodies	<2	Negative	Negative: <20; strong positive: >59
ANA screen with reflex to titer	Negative	Negative	Negative
Anti-ds DNA antibody	2	Negative	Negative ≤4

Case 3

A 76-year-old woman diagnosed with SLE on HCQ of less than 5 mg/kg dose for more than 10 years, history of venous thrombosis and atrial fibrillation on anticoagulation, and hypertensive glomerulonephropathy presented for a routine visit. Her previous autoimmune serology was notable for a positive ANA 1:1280 titer and elevated dsDNA, elevated C-reactive protein (CRP), and negative antiphospholipid antibodies. She reported occasional sicca symptoms and recent intentional weight loss of 11 pounds in four months. Laboratory findings, as mentioned in Table 3, showed renal function with an estimated glomerular filtration rate (eGFR) of 57-51 ml/min/1.73 m^2^ for the past year, normal liver enzymes, mild anemia, and normal erythrocyte sedimentation rate, complement, and dsDNA levels. Her QTc from an ECG more than six months ago is within normal limits (446 ms). Her ophthalmologic examination more than a year ago did not show maculopathy. Based on her recent weight changes, her HCQ dosing was 5.7 mg/kg. HCQ level was obtained, and HCQ was subsequently reduced to less than 5 mg/kg dosing. HCQ level came back with 2,100 ng/ml. HCQ was held for a month and was resumed at 200 mg daily, after which the HCQ level will be repeated after two months.

**Table 3:** Laboratory values of the patient in case 3, notable for a positive ANA 1:1280 titer, eGFR of 51 ml/min/1.73 m2, normal liver enzymes, mild anemia, and normal erythrocyte sedimentation rate, complement, and dsDNA levels; reference values were included. eGFR: estimated glomerular filtration rate; TSH: thyroid-stimulating hormone; ANA: antinuclear antibodies; Anti-ds DNA antibody: anti-double-stranded DNA antibody

Laboratory	Value	Interpretation	Reference range
Leukocytes	5.87	Normal	4.5-11×10^3^/cm^3^
Hemoglobin	11.5	Low	12.0-16.0 g/dl
Platelets	198	Normal	140-440×10³/µL
Blood urea nitrogen	13	Normal	6-22 mg/dl
Creatinine	1.12	High	0.51-0.95 mg/dl
eGFR	51	Low	*≥*60 ml/min/1.73 m^2^
Aspartate transaminases	26	Normal	14-33 IU/l
Alanine transaminases	20	Normal	10-42 IU/l
Albumin	4	Normal	3.8-4.9 g/dl
Hydroxychloroquine level	2,100	High	750-1200 ng/ml
Erythrocyte sedimentation rate	6	Normal	≤20 mm/h
TSH	0.76	Normal	0.270-4.200 mIU/l
ANA titer and pattern	1:1280	High	Negative
Anti-ds DNA antibody	<1	Negative	Negative ≤4
Complement C3	129	Normal	83-193 mg/dl
Complement C4	35	Normal	15-57 mg/dl

## Discussion

We presented two cases with catastrophic adverse events from HCQ and one case of supratherapeutic HCQ whole blood level within the adequate actual weight-based dosing threshold. These three cases illustrate HCQ toxicity despite adherence to standard HCQ dosing, supporting the claim that current monitoring guidelines for HCQ are insufficient. Abrupt discontinuation of HCQ is done at the earliest signs of toxicity and blood levels above the therapeutic range. Traditionally, HCQ monitoring has primarily involved periodic ophthalmologic examinations and adherence to actual weight-based dosing guidelines. However, there are cases without signs of HCQ toxicity that could occur even with supratherapeutic blood levels of HCQ. In the last case, weight-based dosing fails to account for individual variations in drug metabolism and clearance, such as subtle changes in eGFR and acute/subacute weight changes, potentially leading to suboptimal or supratherapeutic dosing with increased risks for systemic toxicity and potential detrimental adverse events as an impending consequence.

Pharmacodynamics, pharmacokinetics, and mechanism of action

HCQ has a chemical formula of C18H26ClN3O [1]. It is primarily absorbed in the upper intestinal tract after a 200 mg oral dose of HCQ, with a bioavailability of 67-74%. HCQ has a large volume of distribution, around 5.5 liters from blood and 44,000 liters from plasma. 

HCQ's high lipophilic properties, along with its high pH and lysosomotropism, allow it to pass through cell membranes. This leads to its accumulation in the lysosomes, which subsequently inhibits endosomal TLR activation and cyclic guanosine monophosphate-adenosine monophosphate (GMP-AMP) synthase-stimulator of interferon genes (STING) activity, crucial for its therapeutic benefits. Moreover, its increased lipophilicity results in widespread tissue sequestration in the liver, kidney, and melanin-rich tissues but not significantly in adipose tissue. However, some studies reported increased HCQ toxicity risk and blood levels in patients with higher body mass index (BMI) and fat composition associated with dosing based on actual body weight [12]. Obesity, defined as BMI ≥30 kg/m^2^, leads to an increase in the volume of distribution and prolongs drug elimination half-life, causing prolonged washout and increased accumulation and exposure [13,14]. HCQ's high distribution into tissues possibly contributes to its wide range of organ toxicity [3]. One prospective cohort study reported that patients with obesity on standard actual body weight HCQ dosing showed higher HCQ blood concentrations with corresponding increased retinopathy risk compared to non-obese patients at the same mg/kg dose [13-15]. Hence, according to the 2016 AAO guidelines for short, obese patients, ideal body weight should be used for HCQ dosing rather than actual body weight [12]. 

Several other mechanisms of HCQ in disrupting cellular functions are enzymatic inhibition and inhibition of apoptosis, autophagy, natural killer cells, and cytokine production. Historically, its half-life is believed to take 40-50 days in the blood, while more recent studies show its elimination half-life could be about five days [1]. This long terminal elimination half-life could be related to significant tissue binding and uptake of HCQ rather than its drug clearance [1]. It takes around 3-4 months to attain a steady-state concentration. 

In addition, HCQ binds to albumin and other serum proteins in plasma and is metabolized through the liver through N-dealkylation by cytochrome P450 enzymes (CYP3A4, CYP2C8, CYP2D6) into its active (desethylhydroxychloroquine) and inactive (desethylchloroquine and bidesethylchloroquine) metabolites [1,3,16]. However, these cytochrome P450 enzymes are altered with impairment in organ function [16]. Thus, in cases of reduced hepatic reserve, such as with preexisting hepatic disease, rare cases of hepatotoxicity, including fulminant and acute toxic hepatitis and markedly elevated transaminase levels even up to 10-fold, could occur in up to 50% of patients with underlying liver disease [17-19]. Its mechanism is not well established, but could be due to dose-dependent changes in hepatic blood flow with increased HCQ bioavailability leading to higher drug levels, an increase in HCQ exposure and accumulation, and delayed and diminished drug clearance [16]. In addition, coexisting illnesses such as porphyria cutanea tarda could increase the risk for acute hepatotoxicity [20]. This necessitates dose adjustments and monitoring of liver function in patients with an increased risk for liver damage, such as taking concomitant hepatotoxic medications, or with a decreased hepatic reserve, such as elderly and malnourished patients, with immediate HCQ withdrawal upon early signs of toxicity [17,21]. 

On the other hand, clearance of HCQ is through renal elimination by almost 50%, compared to around 20% of the dose being excreted unchanged in the urine [3,21]. A decrease in renal function increases the bioavailability of HCQ and subsequently its side effects, and renal failure leads to sub-optimum HCQ levels [1,16]. HCQ also passes the placenta and is found to have equal serum concentrations for both fetus and mother. HCQ is continued and considered safe during pregnancy and breastfeeding, with lower concentrations around 0.2 mg/kg/day in the maternal milk [1]. 

Toxicity

Ocular Toxicity

Ocular toxicity could range from damage involving the cornea, such as vortex keratinopathy or retina with progressive perifoveal annular scotoma [22-24]. Previous studies mentioned an incidence of retinal toxicity from 0.38% to as high as 7.5% with chronic HCQ use of at least five years' duration [24]. These retinal changes involve degenerative changes of photoreceptors in the pigment epithelium and could advance to late-stage findings of classic bull's eye maculopathy with retinal depigmentation [2,22]. Bull's eye maculopathy or classic parafoveal retinopathy is described when it appears in a target-like or "bull's eye" pattern on fundoscopy or imaging. This pattern of lesion shows a well-demarcated concentric zone of atrophy or hypopigmentation surrounding a zone of granular hyperpigmentation of the parafoveal area [23]. In contrast, Asians present with a more pericentral maculopathy without the classic parafoveal retinopathy [24].

Retinopathy increases with cumulative exposure, including higher daily doses and longer duration of HCQ use, from about 1-2% in the first 10 years to 20% for more than 20 years of HCQ use [15,25]. However, the report of retinopathy even after two years was also observed [15]. Current guidelines on retinal toxicity monitoring for HCQ should include baseline ophthalmologic examination, including fundus photography for all patients started on HCQ [12,25,26]. More modern diagnostic testing, such as spectral-domain optical coherence tomography (SD-OCT) and 10-2 visual field analyzer (VFA), has higher sensitivity for the detection of retinal toxicity [2]. SD-OCT is an interferometric technique utilizing the spectral analysis of the interference fringe pattern for higher-resolution cross-sectional imaging [27]. On the other hand, the 10-2 VFA measures the visual sensitivity in the central 10°, while wider angle thresholds outside this central 10° could be of greater utility in Asian patients through the VFA 24-2 test [24,28]. Other imaging modalities, such as mfERG, enhance spatial resolution with better visualization of retinal dysfunction by simultaneously measuring the electrical activity from multiple retinal areas per eye in a rapid timeframe [29]. Discontinuation of HCQ prevents the further progression of lesions, but cases have been reported in which the damage from retinal toxicity could be irreversible, especially in advanced late stages [2].

*Cardiac* *Toxicity*

In 2017, the FDA approved warnings regarding fatal cardiac effects associated with both acute and chronic HCQ use [5]. Cardiotoxicity from HCQ could include arrhythmias, structural and conduction abnormalities, cardiomyopathy, and even cardiovascular collapse leading to death [4,9,11,19,22]. Conduction anomalies, including atrioventricular and bundle branch blocks, are observed more in the short term than in prolonged HCQ use [2]. The COVID-19 pandemic reemerged issues on HCQ's cardiotoxicity since HCQ was used in the acute setting as an experimental drug. QT prolongation has been reported in 1-10% of patients on HCQ, usually between the third and fifth days of initiation. QT interval represents ventricular repolarization and is measured from the Q wave to the T wave, while QTc is the adjusted QT interval that is more accurate despite changes in heart rate [30]. A prolongation of QTc more than 450 ms in males and more than 470 ms in females predisposes to fatal torsades de pointes, a form of polymorphic ventricular tachycardia. Hooks et al. reported that QTc >470 ms during HCQ treatment was associated with increased mortality risk [5]. HCQ-induced arrhythmias are related to HCQ being structurally similar to class IA antiarrhythmic drugs, specifically quinidine [2,4]. On the other hand, structural abnormalities are also observed with HCQ toxicity. These range from ventricular hypertrophy to ventricular hypokinesia in 12% of the patients on HCQ in one study [2]. The pathologic mechanism is believed to be that HCQ alkalizes the cardiomyocyte lysosomes, causing these structural heart diseases. 

Studies reported that HCQ discontinuation could cause complete recovery of cardiac function in almost 1/3 of patients, while others would require further interventions such as cardiac transplantation. In 2021, the American College of Rheumatology released a white paper on the consensus between rheumatologists, cardiologists, and dermatologists on the utilization of ECG for screening and monitoring cardiotoxicity in patients with high-risk cardiovascular factors on HCQ [31]. These cardiovascular factors include female sex, prolonged duration of HCQ use, severe acute illness, more than one QT-prolonging drugs, older age of greater than 68 years, severe electrolyte imbalances (such as hypokalemia, hypomagnesemia, and hypocalcemia), history of recent and hereditary cardiac disease, alcoholic liver disease, hypothyroidism, obesity, anti-Ro 52 positivity, and diabetes. Other studies also suggest performing a baseline ECG, then daily for 5-7 days within the initiation of HCQ, especially in the acute setting [9]. These high-risk cardiovascular patients should have more frequent monitoring for cardiotoxicity, along with those patients with significant renal comorbidities [4,9,21]. High-risk cardiovascular patients on prolonged HCQ use are also recommended to have echocardiography every two years during treatment for cardiotoxicity screening and monitoring [2]. 

Other Systemic Toxicity

Other toxicity from HCQ includes neuromuscular toxicity, with a prevalence of 10%. This could present with bilateral proximal symmetric muscle weakness, polyradiculoneuropathy, neuropathy due to demyelination, pseudoparkinsonism, and even seizures [5,22]. Cutaneous toxicity is another possible HCQ toxicity, with a prevalence of less than 10-20%, with increased reports in those with a prolonged duration of HCQ use. This toxicity could cause chronic pruritus, skin hyperpigmentation, and depigmentation of the skin, nails, and hair. Discontinuation of HCQ use usually results in the partial resolution of skin abnormalities. Oronasopharynx, audiovestibular, hematologic, gastrointestinal, neuropsychiatric, and renal toxicities are also observed with HCQ toxicity. However, partial to complete reversibility of function depending on the stage changes was detected, and when HCQ is discontinued was described [2,5,22]. Pathohistological biopsies of the heart, kidney, spleen, liver, and testis, among other vital organs in rats, were done in another study showing significant pathologic changes with chronic HCQ use [5]. Thus, it is crucial to monitor HCQ toxicity at any time during its use and even more with prolonged high cumulative dosing and high-risk patients. 

HCQ level monitoring and its clinical relevance

HCQ levels are measured in whole blood, serum, or plasma. Whole blood level, which is at least five times the plasma concentrations, is the preferred sample matrix for HCQ level due to its greater precision, following the pharmacokinetics of the drug [32-34]. It is measured by either liquid chromatography or tandem mass spectrometry [35]. HCQ's half-life can occur from 22 days to more than three months, depending on the dose [4]. Complete absorption of HCQ occurs after oral intake. Maximum HCQ levels are observed 2-6 hours after oral administration and must be considered for the interpretation of results [8]. Alternating HCQ doses (e.g., taking 200 mg twice daily and daily alternately) also decreases the accuracy of HCQ level results. These peak HCQ levels cause the rapid onset of severe symptoms, especially in acute toxicity, which is not effectively removed by hemodialysis [3,8,11]. 

Some studies suggest that the determination of HCQ blood level, which is correlated with patient adherence, is also a good predictor of retinal toxicity [36]. A linear association is found between HCQ blood level and retinal toxicity, but there is no clear threshold where this risk is eliminated. In other studies, there is no clear correlation between blood levels and actual weight-based calculated dosing of HCQ [12,35-37]. One study classified HCQ blood levels as subtherapeutic (<750 ng/ml), therapeutic (750-1200 ng/ml), and supratherapeutic (>1200 ng/ml) [29]. HCQ blood levels less than 1075 ng/ml are associated with higher childhood-onset lupus nephritis flares under a prescribed HCQ dose of 4-5.5 mg/kg/day [7]. Other studies recommended 500-1000 ng/ml as a therapeutic target [6,36,37]. A 58% lower risk of active lupus is found in at least 750 ng/ml HCQ levels and even higher doses up to 1192 ng/ml for anti-thrombosis effects [12]. However, higher, supratherapeutic dosing also poses a narrow therapeutic window, leading to greater risks for retinal toxicity, with most toxicity occurring around 1177-3513 ng/ml HCQ blood levels. 

Hence, more individualized monitoring and lower dosing adjustments for higher-risk populations for HCQ toxicity, including the elderly (at least 60 years old), male population, and patients with higher BMI, prolonged duration of HCQ use (especially after five years of use), and other concurrent medications and comorbidities especially renal and hepatic diseases, should always be considered [15,35]. One dermatology study in 2016 reported utilizing HCQ level monitoring every 2-3 months while increasing HCQ dosage for managing active cutaneous lupus until a desirable blood level target is achieved and disease activity is improved [12]. Other than a few reports, there are no existing guidelines on HCQ dosing recommendations and optimal monitoring intervals by professional societies, leaving it to clinical judgment from providers [12]. Hence, it is critical for developing risk stratification models and algorithms integrating these parameters based on expert consensus and pharmacokinetics (Table 4). A lower dosing based on ideal body weight or adjusted body weight, rather than dosing based on actual body weight and drug level monitoring, might be safer in these special higher-risk patient groups. Complete blood count (CBC), comprehensive metabolic panel (CMP), ECG, and HCQ blood level, if available, are checked at baseline. HCQ blood levels are repeated every 6-12 months and sooner if toxicity is suspected or if levels exceed therapeutic ranges. A balance between optimizing therapeutic HCQ levels for ensuring patient adherence and preventing flares while minimizing clinical toxicity is the ultimate goal for providers.

**Table 4:** Risk stratification for special populations requiring HCQ toxicity monitoring and dose adjustments. QTc: corrected QT interval; HCQ: hydroxychloroquine; G6PD: glucose-6-phosphate dehydrogenase; ECG: electrocardiogram; eGFR: estimated glomerular filtration rate

Parameters	Recommendations
Pediatric and older adults at least 60 years old [15,26,31,35]	Start with the lowest effective dose with monitoring for cumulative toxicity; consider more frequent and vigilant monitoring for retinal toxicity (ophthalmologic)
High body mass index/adiposity [12-15]	Avoid dosing by actual body weight in obesity and use ideal or adjusted body weight dosing instead; obtain HCQ blood levels; consider more frequent and vigilant monitoring for toxicity (ophthalmologic, hepatic)
Liver disease(preexisting, concurrent, or high risk, e.g., concurrent alcohol use disorder) [16-21,31]	Base dosing on ideal or adjusted body weight rather than actual body weight; avoid high doses; monitor laboratory (hepatic panel) tests and signs of toxicity more frequently; caution with other hepatotoxic drugs; consider dose reduction/discontinuation according to evidence of toxicity (retinopathy, cardiomyopathy, or persistent transaminitis)
Renal disease(preexisting, concurrent, or high risk) [1,3,16,21]	Monitor laboratory (renal panel and HCQ blood levels) tests and clinical toxicity more frequently; consider dose reduction and more frequent monitoring, especially for retinal toxicity with eGFR <60
Comedications (tamoxifen, nephrotoxic, hepatotoxic, and QTc-prolonging drugs, cytochrome P450 inhibitors, etc.) [31]	Baseline and periodic review of the medication list; consider dose reduction or alternative therapy if high-risk comedications; closer monitoring for toxicity; avoid polypharmacy when possible
G6PD deficiency [12]	Screen before initiation in high-risk ethnic groups, even with low risk for hemolysis; monitor depending on signs and symptoms with complete blood count and possible hemolysis panel
Abnormal surveillance: Persistently elevated HCQ blood levels (cumulative dose/duration), persistent anemia, agranulocytosis, persistent hypoglycemia, thrombocytopenia, transaminitis, or ECG abnormalities [12]	Monitor depending on signs and symptoms with laboratory tests (complete blood count, comprehensive metabolic panel, possible hemolysis panel, HCQ blood levels) and ECG; consider dose reduction/discontinuation or increased monitoring frequency (including annual/biannual ophthalmic surveillance and not delaying until 5 years or more frequent laboratory tests)

Challenges for adopting HCQ level monitoring

Major barriers to utilizing HCQ levels adequately in clinical practice are due to a lack of assay standardization, validation of sample matrices (serum vs whole blood vs plasma), and comparability of results across laboratories [12,38]. There is also high inter-individual variability of HCQ concentrations within the same dosing regimen, related to several factors including BMI [32,33]. In the United States, the cost is around $50 per HCQ blood level measurement and may not be covered by insurance, which could lead to higher out-of-pocket costs for the patients. 

Despite the reported cases of cardiac and retinal toxicities and potential reversibility if caught early, such as in the above cases, to this time, no major guideline for monitoring HCQ, including a target threshold for therapeutic or toxic HCQ drug levels and frequency/interval of monitoring, has been adopted for clinical use by any society. This further complicates the clinical interpretation of HCQ level results [38]. However, several studies reported that monitoring HCQ blood levels is cost-effective in the long term [34,38]. A 66% decrease in acute care utilization and hospitalization risk, especially among Black and Hispanic populations with SLE, is reported if HCQ blood levels are within the 750-1,200 ng/ml range [38]. Due to the cases mentioned, we started utilizing HCQ whole blood level monitoring (CBC, hepatic and renal function) every 3-6 months depending on risk factors as part of our patients' routine toxicity laboratory panel with a therapeutic target between 750 and 1000 ng/ml HCQ levels to monitor compliance and adequate dosing for HCQ in our institution based on other studies [6,35-38]. 

## Conclusions

Traditionally, routine HCQ monitoring has involved periodic ophthalmologic examinations and adherence to weight-based dosing guidelines. This case series highlights the limitations of the current standard of practice following weight-based HCQ dosing with reported toxicities in our institution and strengthens the critical gap in current monitoring strategies for HCQ. Up to this date, there are no universally accepted guideline thresholds or consensus on HCQ blood or serum levels for careful HCQ monitoring in clinical practice. Therefore, we strongly advocate for consensus on utility and implementation of widespread adoption of serial routine HCQ level monitoring upon HCQ initiation and in guiding dose adjustments to avoid unnecessary escalation of immunosuppressive therapy. Constant dose reevaluation should be done, based on individualized risk factors, such as renal and hepatic impairment, older age, significant weight changes and obesity, and concomitant drug use, among others, to monitor HCQ toxicities that could still be reversible with immediate drug discontinuation. HCQ blood level monitoring could serve as a proactive adjunct to existing ophthalmologic surveillance and ECG monitoring. Collaborative efforts are needed to establish standardized protocols for HCQ level monitoring, including frequency and interval for testing, clinical interpretation of results, and adjustment of dosing regimens based on risk factors. Professional societies and regulatory bodies are pivotal in advocating these guidelines and promoting their integration into clinical practice. Future comparative studies should be implemented to evaluate the efficacy of HCQ blood level monitoring concerning the timing and frequency of interval monitoring and adequate therapeutic and toxic HCQ levels, in minimizing and detecting early toxicities while optimizing the benefits of HCQ.
